# Predicting population-level vulnerability among pregnant women using routinely collected data and the added relevance of self-reported data

**DOI:** 10.1093/eurpub/ckae184

**Published:** 2024-11-27

**Authors:** Joyce M Molenaar, Ka Yin Leung, Lindsey van der Meer, Peter Paul F Klein, Jeroen N Struijs, Jessica C Kiefte-de Jong

**Affiliations:** Population Health and Health Services Research, Centre for Public Health, Healthcare and Society, National Institute for Public Health and the Environment (RIVM), Bilthoven, the Netherlands; Department of Public Health and Primary Care/Health Campus The Hague, Leiden University Medical Centre, The Hague, the Netherlands; Department of Statistics, Data Science and Modelling, National Institute for Public Health and the Environment (RIVM), Bilthoven, the Netherlands; D epartment of Obstetrics and Gynaecology, Erasmus MC, University Medical Centre, Rotterdam, the Netherlands; Population Health and Health Services Research, Centre for Public Health, Healthcare and Society, National Institute for Public Health and the Environment (RIVM), Bilthoven, the Netherlands; Population Health and Health Services Research, Centre for Public Health, Healthcare and Society, National Institute for Public Health and the Environment (RIVM), Bilthoven, the Netherlands; Department of Public Health and Primary Care/Health Campus The Hague, Leiden University Medical Centre, The Hague, the Netherlands; Department of Public Health and Primary Care/Health Campus The Hague, Leiden University Medical Centre, The Hague, the Netherlands

## Abstract

Recognizing and addressing vulnerability during the first thousand days of life can prevent health inequities. It is necessary to determine the best data for predicting multidimensional vulnerability (i.e. risk factors to vulnerability across different domains and a lack of protective factors) at population level to understand national prevalence and trends. This study aimed to (1) assess the feasibility of predicting multidimensional vulnerability during pregnancy using routinely collected data, (2) explore potential improvement of these predictions by adding self-reported data on health, well-being, and lifestyle, and (3) identify the most relevant predictors. The study was conducted using Dutch nationwide routinely collected data and self-reported Public Health Monitor data. First, to predict multidimensional vulnerability using routinely collected data, we used random forest (RF) and considered the area under the curve (AUC) and F1 measure to assess RF model performance. To validate results, sensitivity analyses (XGBoost and Lasso) were done. Second, we gradually added self-reported data to predictions. Third, we explored the RF model’s variable importance. The initial RF model could distinguish between those with and without multidimensional vulnerability (AUC = 0.98). The model was able to correctly predict multidimensional vulnerability in most cases, but there was also misclassification (F1 measure = 0.70). Adding self-reported data improved RF model performance (e.g. F1 measure = 0.80 after adding perceived health). The strongest predictors concerned self-reported health, socioeconomic characteristics, and healthcare expenditures and utilization. It seems possible to predict multidimensional vulnerability using routinely collected data that is readily available. However, adding self-reported data can improve predictions.

## Introduction

A strong foundation during the first thousand days of life, which span from conception till a child’s second birthday, can positively impact health and development in later life and across generations [1, 2]. Adverse experiences and exposures can influence the health of parents themselves, but can also be transmitted to their children, which, as these children grown into adulthood and potentially become parents themselves, leads to new cycles of adversity [2]. In order to prevent health inequities and break the intergenerational cycle, it is important to recognize and address vulnerability during the first thousand days [1–3]. This is also a focus in the Dutch national action programme Solid Start (in Dutch: Kansrijke Start) [4]. The concept of vulnerability is often used to describe subgroups with increased risks to adverse health outcomes or limited access to healthcare. In short, vulnerability encompasses a multifaceted and dynamic process in which diverse stressors at the individual or contextual level can serve as risk factors, whereas protective factors have the potential to mitigate or prevent vulnerability [5–8]. Examples of risk factors encompass unemployment or stress, while examples of protective factors include a strong social network or effective coping skills.

The concept of vulnerability and its scope has garnered increasing attention among providers and policymakers who strive to enhance the provision of care and support during the first thousand days of life [4, 9, 10]. In daily care, a common understanding between professionals from the medical and social sectors on the characteristics of high-risk individuals can foster mutual understanding and improve cross-sectoral collaboration [9]. At national and local policy levels, drawing attention to the prevalence, geographical distributions, and trends in vulnerability can support policy monitoring and prioritization. These insights not only foster a sense of urgency, but also enhance the conversation between different stakeholders, and facilitate vision formulation and intervention prioritization [11].

This study extends our prior research to predict population-level vulnerability among pregnant women. Our previous study highlighted the significance of considering both risk and protective factors, particularly in the context of adverse outcomes [12]. Through Latent Class Analysis (a data-driven technique to identify subgroups with similar characteristics), we identified five groups of pregnant women with different social risk and protective factors to vulnerability prior to pregnancy. Women in the ‘multidimensional vulnerability’ group shared multiple risk factors across several domains (e.g. psychosocial, medical, and socioeconomic), lacked protective factors, and were most at risk of adverse outcomes such as premature birth and caesarean section. Having risk factors in a single domain (e.g. socioeconomic) was not necessarily associated with adverse outcomes. This previous study utilized both routinely collected observational data and self-reported data on health, well-being, and lifestyle of the Public Health Monitor 2016 (PHM-2016) to predict multidimensional vulnerability [12]. Using the PHM-2016 resulted in a smaller subset of the total Dutch pregnant population. Hence, the prevalence of multidimensional vulnerability across the entire population of pregnant women at a national level remains unknown, and it is unclear whether this can be assessed using solely routinely collected observational data and what the added value of self-reported data is. Moreover, we lack an understanding of the strongest predictors for population-level vulnerability.

Mapping out the percentage of multidimensional vulnerability among pregnant women in the Netherlands and its predictors is relevant for risk stratification. In population health management, this is an essential initial step to tailor (preventive) actions to the needs of specific risk groups to enhance population health [13, 14]. Such stratification commonly relies on routinely collected data [15], offering advantages such as widespread availability, reduced practitioner burden, time, and costs [16, 17]. Moreover, the longitudinal and systematic approach facilitates comparisons over time [16, 17]. However, it is important to empirically evaluate whether routinely collected data is sufficient for risk stratification for high-risk groups. In addition, there is a potential for improvement in predicting multidimensional vulnerability at population level by incorporating self-reported health, well-being, and lifestyle data. For example, studies indicate that self-reported health and vulnerability correspond to or complement clinical measures in predicting adverse health outcomes [18–22]. Yet, the impact of adding self-reported data next to routinely collected data in predicting vulnerability remains unexplored.

This study has three objectives. First, to assess the feasibility of accurately predicting multidimensional vulnerability during pregnancy at population level using solely routinely collected observational data. We use the predictions to report on the prevalence and spatial variation of multidimensional vulnerability during pregnancy at population level in the Netherlands. Second, to identify whether self-reported data on health, well-being, and lifestyle could improve those predictions with routinely registered data. Third, to identify the predictors that have the most significant impact on the classification of multidimensional vulnerability.

## Methods

### Data sources

This study employed data from DIAPER (Data InfrAstructure for ParEnts and ChildRen) [17]. DIAPER integrates individual-level, routinely collected observational data from various national registries, including Perined and Statistics Netherlands. Perined collects routine care data on pregnancy, birth, and the first 28 days after birth from midwives, gynaecologists, and paediatricians [23]. Statistics Netherlands collects data about social issues, including health, welfare, income, education, and labour [24, 25]. To enrich DIAPER, self-reported data on health, well-being, and lifestyle of the PHM-2016 were included for those who participated in the PHM [26]. The PHM is a health survey conducted every 4 years among a varying sample of Dutch adults aged 19 years and older (about 450.000 in 2016).

### Study population

The study population consisted of 4172 unique women with a pregnancy and childbirth in 2017 or 2018 who participated in the PHM-2016 prior to pregnancy. Details on selecting the study population are described elsewhere [12]. To illustrate the prevalence and spatial variation of multidimensional vulnerability at national level, all unique registered pregnancies in Perined from 2017 to 2021 were considered (*n* = 807.904) [17]. Missing data were imputed through Multiple Imputation using Chained Equations (MICE), leading to five imputed datasets [27].

### Independent variables

Analogous to our previous study, we included 42 variables in the predictive models [12]. Details on these variables have been described in Supplementary Appendix S1 of our previous study [12]. The first category in each variable denotes the risk factor for vulnerability.

Of those 42 variables, 31 variables concerned routinely collected data available for all pregnant women in DIAPER (*n* = 807.904). Those included individual characteristics (age, ethnicity, parity, asylum seeker status), socioeconomic characteristics (educational level, household income, socioeconomic position by occupational status, debts and payment arrears, permanent employment contract, and full-time employment contract), household characteristics (type of household, marital status, dissolution of marriage, household size, and youth support utilization), healthcare expenditures and utilization (total healthcare expenditures, general practitioner’s (GP) expenditures, hospital expenditures, medication use, and addiction-related care utilization), psychosocial characteristics (mental healthcare utilization, mild intellectual disability), life events (crime suspect, crime victim, having been detained, frequent moving, loss of a family member), living conditions (home ownership, motorized vehicle ownership, proximity to GP office, liveability neighbourhood).

The other 11 variables were derived from the PHM-2016 and consequently only available for 4172 individuals. These variables included lifestyle factors (smoking, alcohol use, physical activity, body mass index (BMI)), self-reported health (perceived health status, long-term illness, restricted by health), psychosocial characteristics (risk of depression or anxiety disorders, loneliness, feelings of control over life) and socioeconomic characteristics (insufficient financial resources).

### Outcome: multidimensional vulnerability

The outcome measure is multidimensional vulnerability, as derived from our previous study [12]. Women classified into the ‘multidimensional vulnerability’-class share a combination of multiple risk factors to vulnerability in several domains and lack protective factors. It is not a straightforward equation and risk factors vary across individuals. Most present risk factors include not having an income or receiving benefits, rental housing, high GP healthcare expenditures, long-term illness, negative self-perceived health, and elevated risks of feeling lonely, depressed, or anxious.

We added the variable multidimensional vulnerability to the dataset of 4172 individuals. All women who were previously assigned to the multidimensional vulnerability class were classified as ‘yes’ (*n* = 249) and women in all other classes as ‘no’ (*n* = 3923).

### Statistical analyses

To assess whether it is feasible to predict multidimensional vulnerability during pregnancy using solely routinely collected data at population level (objective 1), we employed random forest (RF). RF is a machine learning method for regression and classification that operates through the construction of multiple decision trees [28]. The method makes no assumptions about data distribution and works well with the number of individuals in our dataset relative to the number of variables. Sensitivity analyses were conducted using XGBoost and Lasso for validation (see Supplementary Appendix S1).

We sought the optimal model using the area under the curve (AUC) and F1 measure [29]. The AUC, ranging between 0.5 (random) and 1.0 (perfect model), illustrates the ability of the model to distinguish between those with and without multidimensional vulnerability. Due to our imbalanced dataset with relatively few cases of multidimensional vulnerability, we calculated F1 measures to focus on correct predictions of vulnerability [29]. The F1 measure balances precision, also known as positive predictive value (i.e. proportion of correct predictions out of all predicted as vulnerable) and recall/sensitivity (i.e. proportion of individuals with vulnerability correctly predicted as vulnerable by the model). We treated both elements as equally important. A perfect score means the model can identify *all* positive cases while also identifying *only* positive cases (instead of assigning those without vulnerability incorrectly to the vulnerability class). We additionally report on specificity (i.e. proportion of correct negative predictions out of all without vulnerability) and the confusion matrices showing true/false positives and true/false negatives. In model development, we used default hyperparameters settings in the R-packing ‘ranger’ [30], as these typically perform well. We used nested cross-validation to choose the threshold probability for classifying multidimensional vulnerability into ‘yes’ and ‘no’ and to assess model performance [31]. This involved splitting the dataset in an outer loop (six folds of train–test combinations) and inner loop (five train–validate combinations), detailed in Supplementary Appendix S1. The final RF model can be utilized for predicting outcomes on new datasets. Being the best performing model, it was also used to report on the prevalence and spatial variation of multidimensional vulnerability during pregnancy from 2017 to 2021. We computed percentages for both national and municipality levels in the five imputed datasets and conducted an additional complete case analysis at national level for comparison. Municipality-level results were visualized on a map of the Netherlands.

Next, to identify if self-reported data on health, well-being, and lifestyle could improve predictions with solely routinely collected data (objective 2), we gradually added self-reported data from the PHM-2016 to the RF model. Using the previous six train–test combinations, we calculated average F1 measures for different variable sets; (1) solely routinely collected data (baseline, 31 variables); (2) baseline combined with one varying PHM-2016 variable (comprising 32 variables); (3) baseline combined with two varying PHM-2016 variables (comprising 33 variables); and (4) baseline combined with all PHM-2016 variables, representing a potential optimum (42 variables). Comparing average F1 measures for each combination helped identify which PHM-2016 variables enhanced model performance.

To identify which variables were most important in model predictions (objective 3), we assessed variable importance in the final RF model with and without PHM-2016 data. Variable importance was measured using out-of-bag observations, explained in Supplementary Appendix S1. This process yields a ranking of variable importance [32]. As sensitivity analyses, we checked the permutation importance and partial dependence plots (PDPs), explained in Supplementary Appendix S1.

### Ethical approval

The Clinical Expertise Centre of the National Institute for Public Health and the Environment confirmed that our study was not subject to the Dutch Medical Research involving Human Subjects Act (WMO; reference number: VPZ-574).

## Results

### Study population

The study population comprised 4172 women (Supplementary Appendix S1). Approximately 42.1% of these women were nullipara, 4.6% had a low income and 6.0% had a low educational level. In comparison to all women with unique pregnancies between 2017 and 2021 (*n* = 807.904), the distribution regarding most variables was comparable, but differences were found for variables such as income, educational level, and ethnicity. Among the 4172 women, there was generally a lower incidence of the risk factors.

### Predictions with routinely collected data

The RF model which included the routinely collected data obtained an average AUC of 0.98 (see Table 1). Such a high AUC implicates that the model sufficiently distinguishes between those with and without multidimensional vulnerability. The F1 measure had an average of 0.70, indicating that the model is able to correctly predict cases of multidimensional vulnerability, but that there are also cases missed as well as women incorrectly assigned to the vulnerability class (see Table 1). Supplementary Appendix S2 presents the selected hyperparameters and thresholds and the results of the separate folds. Results were consistent with those of XGBoost and Lasso (Supplementary Appendix S2).

**Table 1.** Results of the RF and the sensitivity analyses

	Metrics					Confusion matrices for best fold
	Mean from five-fold cross-validation (SD)					Number in each category
	AUC	F1 measure	Precision	Recall/sensitivity	Specificity	
Random forest	0.98 (0.00)	0.70 (0.03)	0.74 (0.06)	0.66 (0.04)	0.98 (0.00)	30 (TP) 14 (FN) 6 (FP) 645 (TN)
XGBoost	0.98 (0.00)	0.68 (0.04)	0.70 (0.02)	0.67 (0.08)	0.98 (0.00)	34 (TP) 13 (FN) 10 (FP) 638 (TN)
Lasso regression	0.98 (0.01)	0.68 (0.04)	0.67 (0.07)	0.70 (0.07)	0.98 (0.01)	32 (TP) 11 (FN) 12 (FP) 640 (TN)

AUC, area under the curve; FN, false negative; FP, false positive; TN, true negative; TP, true positive.

Results based on analyses among study population of 4172 women.

**Figure 1.**
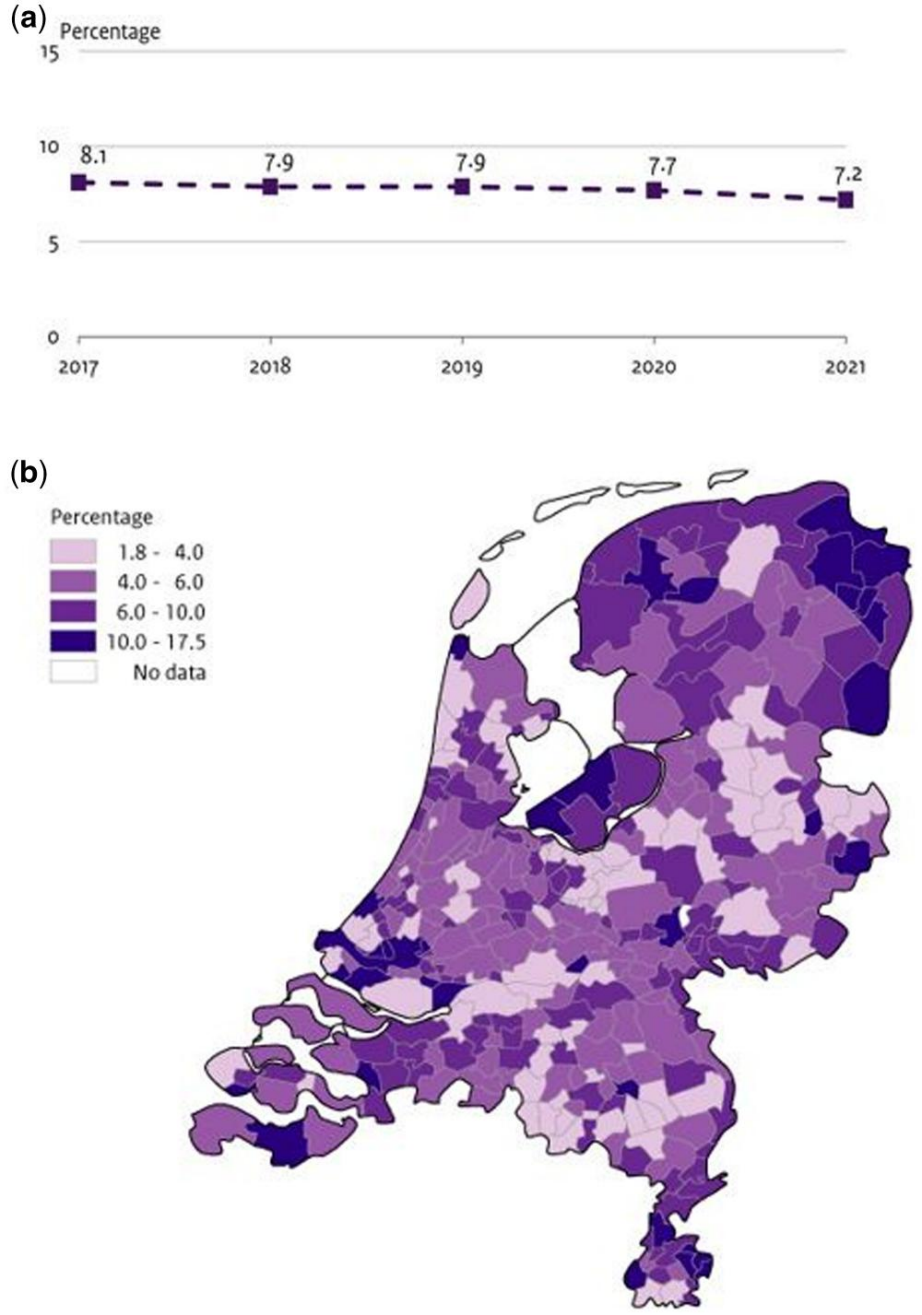
Percentage of multidimensional vulnerability during pregnancy in The Netherlands. (a) The nationwide percentages during the years 2017 to 2021, based on the RF model using routinely collected data prior to pregnancy. (b) A heatmap visualizing the geographical distribution at municipality level, for all pregnancies from 2017 till 2021. A darker colour indicates a higher percentage of vulnerability. Results based on analyses among all unique pregnancies from 2017 to 2021 (*n* = 807.904).

The percentage of individuals with multidimensional vulnerability during pregnancy in the Netherlands was 8.1 in 2017 and decreased to 7.2 in 2021, as derived from the RF model (Fig. 1a). The percentages were slightly higher for XGBoost and lasso (respectively 8.0% and 9.1% in 2021), but showed a similar decreasing trend, as printed in Supplementary Appendix S2. Supplementary Appendix S2 additionally shows the complete case analysis.

Figure 1b visualizes the geographical distribution of multidimensional vulnerability during pregnancy in the Netherlands over the years 2017–2021, based on predictions of the RF model. There are differences between municipalities, with percentages ranging from 1.8% to 17.5%.

### Adding self-reported data to predictions

**Figure 2.**
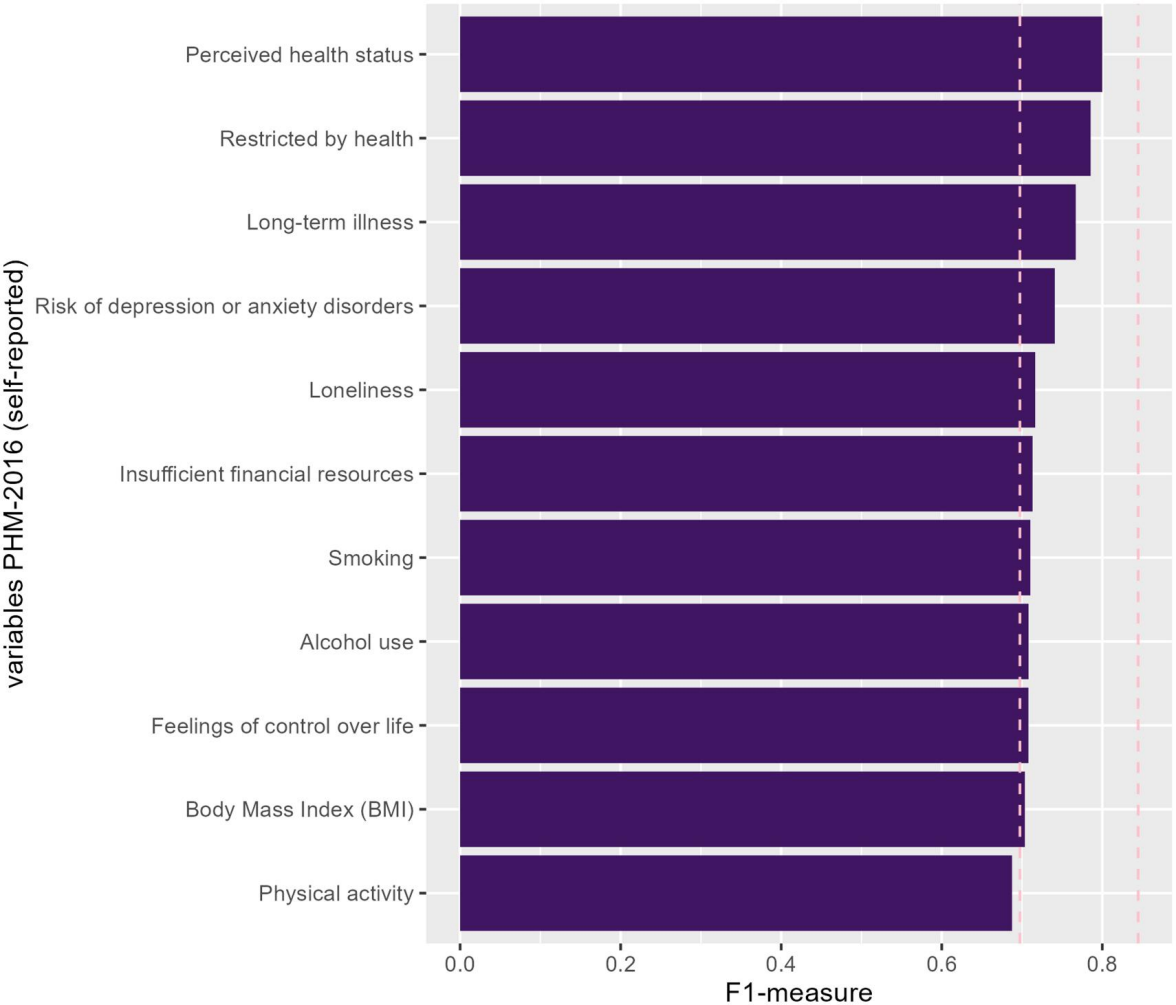
Variables on self-reported health, well-being, and lifestyle added to the RF model with solely routinely collected data. The vertical lines show the average F1 measures. Results based on analyses among the study population of 4172 women.

The baseline F1 measure (using routinely collected data; 31 variables) was 0.70 and the potential optimum (using both routinely collected data and all self-reported data of the PHM-2016; 42 variables) was found to be 0.83, shown as vertical lines in Fig. 2. Including self-reported variables improved the performance of the RF models with solely routinely collected data. Especially self-reported data on ‘perceived health status’ (average 0.80) and ‘restricted by health’ (0.79) improved the model’s performance, but also ‘long-term illness’ (0.77) and ‘risk to depression or anxiety disorders’ (0.74). Others had little impact or slightly decreased performance, such as physical activity. Supplementary Appendix S2 presents the results of adding two varying self-reported variables. This further improved the performance of the model.

### Variable importance

**Figure 3.**
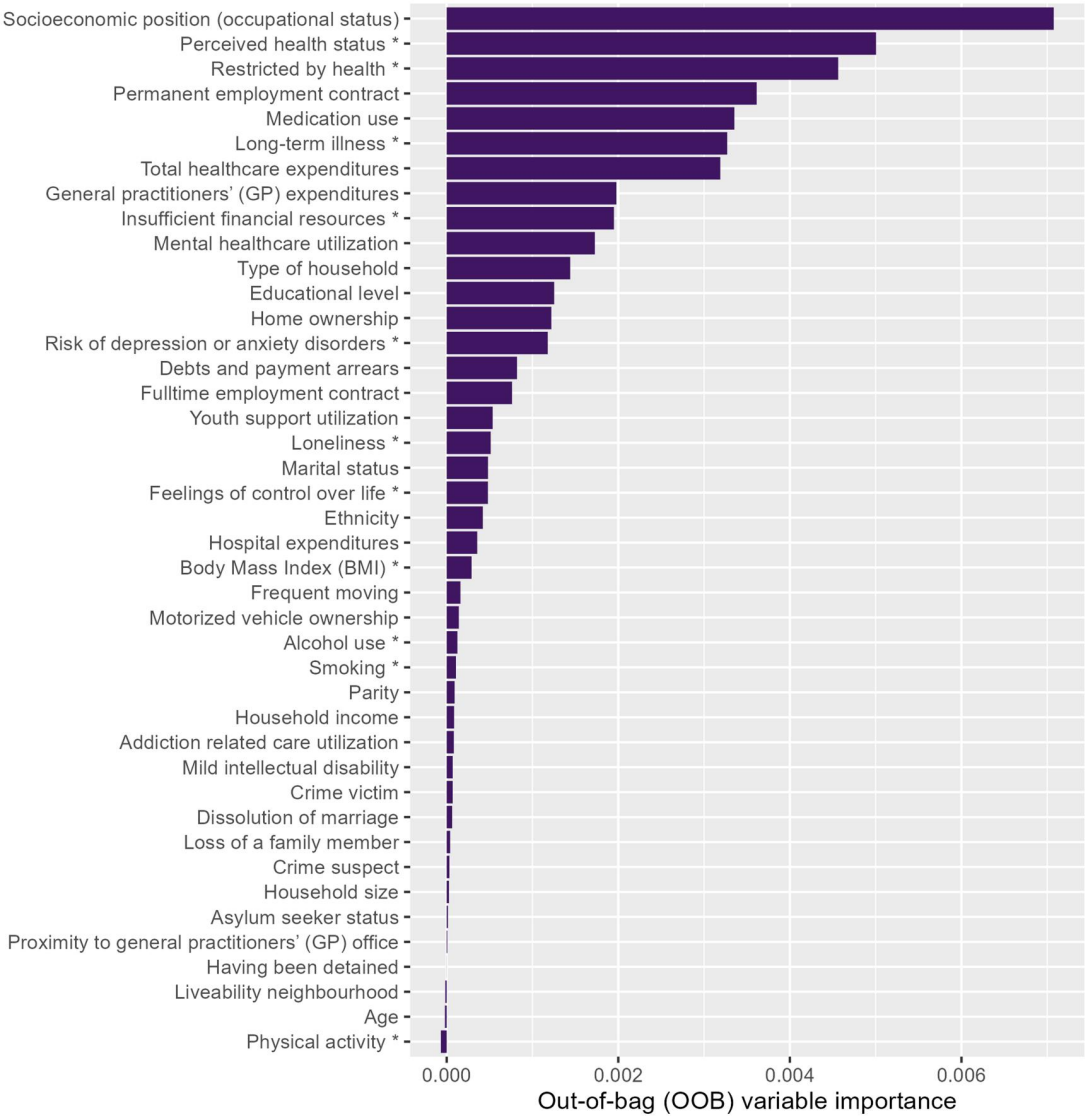
Variable importance ranking of the RF model for ‘multidimensional vulnerability’. The set of 42 variables used for classification are ordered from high to low importance. The length of a line represents the importance of a particular variable on the model’s predictions. Self-reported data of the PHM-2016 is indicated with an asterisk (*). Results based on analyses among the study population of 4172 women.

Figure 3 shows the variable importance of all 42 variables. Top seven predictors for multidimensional vulnerability during pregnancy were: ‘socioeconomic position (occupational status)’, ‘perceived health status’, ‘restricted by health’, ‘permanent employment contract’, ‘medication use’, ‘long-term illness’, and ‘total healthcare expenditures’. Out of these seven variables, which represent both risk and protective factors, three concern self-reported health, two concern socioeconomic characteristics and two relate to healthcare expenditures and utilization. Related to financial status, self-reported ‘insufficient financial resources’ was ranked higher compared to the routinely collected ‘household income’ and ‘depts and payment arrears’. Likewise, self-reported ‘perceived health status’ and ‘feeling restricted by health’ were ranked higher than ‘medication use’ and ‘total healthcare expenditures’. We found the opposite for psychological characteristics: routinely collected ‘mental healthcare utilization’ was ranked higher than self-reported ‘risk of depression or anxiety disorders’ or ‘loneliness’. However, differences were small.

The permutation importance ranking (Supplementary Appendix S2) yielded comparable results, although ‘mental healthcare utilization’ and ‘GP expenditures’ were ranked slightly higher. Supplementary Appendix S2 additionally shows the rankings without self-reported data, using solely routinely collected data (31 variables).

## Discussion

This study provides insight into predicting multidimensional vulnerability during pregnancy at population level in the Netherlands using pre-pregnancy routinely collected data and the relevance of additional self-reported data on health, well-being, and lifestyle. Based on our results, it seems reasonably feasible to predict multidimensional vulnerability using solely routinely collected data, since the RF model could distinguish between those with and without multidimensional vulnerability and was able to correctly predict multidimensional vulnerability in many cases. However, we found that adding self-reported data improved model performance. Out of the seven strongest predictors to multidimensional vulnerability in our dataset, three concerned self-reported health, two concerned socioeconomic characteristics, and two related to healthcare expenditures and utilization.

Using solely routinely collected data to predict multidimensional vulnerability appears feasible, but several women were wrongly assigned to the vulnerability class, and other cases were missed. The crucial concern is whether the model achieved adequate performance, prompting consideration of using this readily available routinely collected data versus acquiring self-reported data on experienced health. Both data sources have advantages and disadvantages and may be used for different purposes. Using routinely collected data is relatively easy, accessible, and time-efficient. This pragmatic approach recognizes that not all data are available and can be utilized, analysed, and interpreted. However, it is less accurate which might mainly affect those missed by the model. Considering all relevant factors by using additional self-reported data leads to better predictions. However, this has numerous implications and inherent challenges, including increased burden to practitioners, time, and costs. Based on our study, we consider routinely collected data sufficient for policy monitoring of multidimensional vulnerability at population level. It can offer insight into its scope and development over the years and help identify municipalities and neighbourhoods characterized by increased vulnerability, enabling tailored (preventive) measures for efficient budget allocation. Simultaneously, we agree with previous scholars that applying vulnerability in a dichotomous way is challenging as the concept is multilayered, contextualized, and dynamic, requiring caution to avoid over-inclusion or exclusion of individuals [33, 34]. Our previous study [12] revealed a greater array of vulnerability groups, with women having risk factors within one specific domain and protective factors in others. We must not overlook these and other intermediary and personal, contextual forms of vulnerability. Our predictive RF model was not intended for application in individual predictions and individual decision-making but meant for risk stratification on a population level. Because risk assessment is not straightforward, we consider routinely collected data by itself unsuitable for individual predictions, given that it insufficiently accounts for protective factors and coping strategies at an individual level, among others. We believe that an open conversation with (future) parents about their experienced health and well-being is indispensable to better understand their context and needs. It is essential that this is accompanied by a trusting relationship, and appropriate follow-up steps, preventing stigmatization, simplification, and harm [20, 33, 35]. Given the added value of self-reported data, however, we suggest to explore how perceived health can be systematically included into screening guidelines and care registries for professionals, to enhance the provision of personalized care and support while further improving population-level predictions in the future.

In our study, adding self-reported data led to better model performance and self-reported health indicators were found as important predictors to multidimensional vulnerability. Consistent with the psychosocial literature, several subjective measures (e.g. self-reported ‘insufficient financial resources’) outweighed objective measures (e.g. registered ‘income’ and ‘debts and payment arrears’) as predictors in our study. For example, multiple studies reported a stronger link between people’s subjective socioeconomic status (SES) and well-being and physical health compared to objective SES based on income or education [36–39]. Arguably, perceiving your circumstances through the lens of limited resources impacts decision-making and behaviour (e.g. favouring short-term over long-term considerations), increases uncertainties and stress, and thus exacerbates pre-existing vulnerabilities [40–43]. Other studies reported how self-reported health or vulnerability correspond to, outperform, or complement clinical measures in predicting physical health and mortality [18–20]. However, using self-reported health also has its challenges. For instance, it provides little guidance regarding what respondents consider when reporting ‘poor health’ and whether they refer to physical pain, mental well-being, less vitality or other factors [21]. Additionally, people can have diverse perceptions of their health influenced by cultural contexts, social positions, and personal health experiences (e.g. people suffering from the same illness for a longer time may report better levels of health due to various coping and self-management strategies) [22, 44]. Nevertheless, self-reported health seems to be an important measure which can capture components of health or vulnerability that other measures alone cannot.

### Strengths, limitations, and future research

The availability of nationwide data on a wide range of risk and protective factors to vulnerability in many different domains was an important strength of this study. The outcome ‘multidimensional vulnerability’ was also based on 42 variables [12]. Additionally, we conducted several sensitivity analyses, all of which yielded similar results, underscoring the robustness of our model. However, this study also had several limitations, mostly related to the data. One limitation concerns the representativeness of the dataset used to construct and evaluate the predictive models. It is possible that some factors (e.g. asylum seeker status) did not emerge as primary predictors because they were less present among the 4172 women, despite their association with vulnerability and adverse outcomes in the literature [45, 46]. This may have also led to a slight underestimation of the actual percentage of multidimensional vulnerability. Additionally, we missed data on important topics that can contribute to vulnerability such as stress, health literacy, coping skills, and adverse (childhood) experiences including violence. Another limitation is that we insufficiently considered the dynamics around pregnancy in relation to vulnerability, since we merely incorporated data prior to pregnancy that can be subject to change. Future research should take into account that vulnerability can exist prior to pregnancy, but also arise or change during pregnancy, childbirth, or after birth [9]. Also, a consideration of the role of the father or woman’s partner and wider social network could contribute to more insights into vulnerability and better predictions.

## Conclusions

This study shows that it is feasible to predict multidimensional vulnerability at population level using solely routinely collected data. Routinely collected data is readily available for the entire population, thereby providing a robust foundation for longitudinal monitoring and policy formulation at population level. Nevertheless, while predictions are fairly accurate, adding self-reported data is of added value.

## Supplementary Material

ckae184_Supplementary_Data

## Data Availability

We are unable to share the individual data used for this study as data linkage and analysis was conducted within the highly safeguarded Remote Access (RA) platform of Statistics Netherlands. All data within this platform are pseudonymized to ensure data safety and confidentiality. Access to the data from Perined, Statistics Netherlands, and the Public Health Monitor 2016 can be requested from the relevant parties. Key points Considering the combination of both social risk and protective factors related to vulnerability is needed to identify pregnant women at risk of adverse outcomes. Multidimensional vulnerability at population level can be predicted using solely routinely collected data. Using self-reported data in addition to routinely collected data can be relevant to further improve the prediction of multidimensional vulnerability. The strongest predictors to multidimensional vulnerability are related to self-reported health, socioeconomic characteristics, and healthcare expenditures and utilization. Without additional data collection, routinely collected data could provide insight into the prevalence, geographical distribution, and trends in multidimensional vulnerability at population level, which can be used for longitudinal monitoring and the formulation of policies.
