# Supplementary material for: Predicting population-level vulnerability among pregnant women using routinely collected data and the added relevance of self-reported data
Source: Eur J Public Health. 2024 Nov 27;34(6):1210–7. doi: 10.1093/eurpub/ckae184 (PMC11631480; doi:10.1093/eurpub/ckae184)
Supplement: ckae184_Supplementary_Data [file ckae184_supplementary_data.pdf]

Appendices

Appendix A. Methodology

Sensitivity analyses: XGBoost and Lasso regression

XGBoost (extreme gradient boosting) is a machine learning technique that iteratively builds multiple shallow decision trees (1). Similar to RF, it is a flexible algorithm without assuming a functional form. Logistic regression, on the other hand, does assume a strong functional form, i.e. a linear relation between the independent variables and log odds. Logistic regression is a standard approach for binary classification with a long history in literature. The logistic regression analysis was conducted with lasso penalty to shrink coefficients towards zero such that less important variables are left out the model (2).

Nested cross-validation

The three techniques RF, Lasso and XGBoost each have their own set of hyperparameters that need to be chosen for the models. For RF, the default hyperparameter settings in the R-package 'ranger' (3) were used, as these default settings generally yield good performance. The parameter to choose for Lasso (R-package 'glmnet' (4)) was the lambda, which defines the penalty, and for XGboost (R-package 'xgboost' (5)) the number of trees and tree-depth. For Lasso and XGBoost we used cross-validation to choose the hyperparameters. In addition, as the models predict the probability of multidimensional vulnerability, we need to choose the threshold at which all predicted probabilities above that threshold are classified as multidimensional vulnerable 'yes' (and as 'no' below that threshold). To choose the hyperparameters and threshold probability, and finally to assess the performance of the models we used nested-cross validation.

Firstly, the dataset of 4172 women was split into six folds: 5 parts train-set, 1 part test-set (outer loop). Secondly, in the nesting step (inner loop), each train set from the outer loop was again split into five folds: 4 parts train-set, 1 part validation-set. During each split, we made sure that the percentage of multidimensional vulnerability was approximately equal in each part.

Firstly, using the cross-validation of the inner-loop, we chose the hyperparameters: for Lasso, we chose the average lambda across the five validation folds and for XGBoost we selected the hyperparameters for which the average AUC over the five folds was highest. Secondly, using the defined hyperparameters, and the same inner loop, we selected the threshold probability that yielded the highest F1-value on the validation set (averaged over five validation folds). Thirdly, by utilizing both the selected hyperparameters and average optimal thresholds, we calculated the F1-value on the test set of the outer loop that has not been used in selecting hyperparameters and threshold. This three-step process was repeated for the 6 folds of the outer loop, yielding the F1-measure of the model averaged over the 6 test-sets. Using the average optimal thresholds (as well as the defined hyperparameters), we fitted the model one last time on all data. The final model can be utilized for predicting outcomes on new datasets. We used the final RF model in our next analyses.

Variable importance with OOB-observations, including sensitivity analyses

Variable importance was measured with RF in the following way. RF takes a bootstrap sample for every tree that it constructs. The data that are not used in the bootstrap sample are called the out-of-bag (OOB) observations. RF makes a prediction for these OOB-observation based on the tree that is constructed on the bootstrap-sample, leading to an OOB error. Next, to determine the tree-specific importance of a variable, a variable is randomly shuffled (permuted) in the bootstrap sample. In this new variant of the bootstrap sample, a new tree is grown which gives a new OOB error. This OOB error is then compared to the original OOB error. If permuting a variable increases the error, it is considered important as the model relied on it for prediction. Consequently, by permuting a variable and comparing the OOB error rates of the predictions before and after permutation (6), we obtain a measure of variable importance for each variable for a single tree. The OOB-errors increase for each variable are averaged over all trees and compared. The average of all these tree importance values yields the ranking of variables for the model (7).

As sensitivity analyses, we also checked the permutation importance and Partial Dependence Plots (PDP)(8-10). Permutation importance permutes the values of a specific variable in the full dataset (rather than individual trees) to measure the impact on the model's performance. The PDP of each variable provides insight into the direction and strength of the relationship with the dependent variable while holding all other predictors constant. We checked whether the direction of the important variables aligned with their categorization as risk or protective factors.

Appendix B. Results

Table B.1. Characteristics of the study populations (including missing data)

		Original study population *(objective 1, 2 and 3)*	All unique pregnancies 2017 – 2021 *(objective 1)*
	Total n =	4172	807.904
		n (%)	n (%)
**Individual characteristics**			
Age	19-23	306 (7.3)	43797 (5.4)
	24-35	3528 (84.6)	624292 (77.3)
	>35	338 (8.1)	135882 (16.8)
	Missing		*3933 (0.5)*
Ethnicity	Non-Western	420 (10.1)	170968 (21.2)
	Western	343 (8.2)	89970 (11.1)
	Native Dutch	3409 (81.7)	546624 (67.7)
	Missing		*342 (<0.1)*
Parity[a]	Nullipara	1755 (42.1)	355979 (44.1)
	Primipara, multipara	2410 (57.8)	449416 (55.6)
	Missing	*<10 (<0.2)*	*2509 (0.3)*
Asylum seeker status	Yes	39 (0.9)	19582 (2.4)
	No	4133 (99.1)	788322 (97.6)
**Socioeconomic characteristics**			
Educational level	Low	328 (7.9)	80849 (10.0)
	Moderate	1513 (36.3)	289681 (35.9)
	High	2303 (55.2)	340669 (42.2)
	Missing	*28 (0.7)*	*96705 (12.0)*
Household income	Low	202 (4.8)	60682 (7.5)
	Moderate	3348 (80.2)	613979 (76.0)
	High	*591 (14.2)*	103122 (12.8)
	Missing	*31 (0.7)*	*30121 (3.7)*
Socioeconomic position (occupational status)	No income/receiving benefits	532 (12.8)	132780 (16.4)
	Student	82 (2.0)	24988 (3.1)

	Paid work	3502 (83.9)	632685 (78.3)
	Missing	*56 (1.3)*	*17451 (2.2)*
Debts and payment arrears	Yes	45 (1.1)	19357 (2.4)
	No	4127 (98.9)	788547 (97.6)
Insufficient financial resources	Yes	524 (12.6)	
	No	3267 (78.3)	
	Missing	*381 (9.1)*	
Permanent contract	No	1929 (46.2)	433393 (53.6)
	Yes	2243 (53.8)	374511 (46.4)
Full-time contract	No	1925 (46.1)	653069 (80.8)
	Yes	2247 (53.9)	154835 (19.2)
**Lifestyle factors**			
Smoking	Yes	661 (15.8)	
	No	3315 (79.5)	
	Missing	*196 (4.7)*	
Alcohol use	Yes (excessive)	418 (10.0)	
	No	*3503 (84.0)*	
	Missing	*251 (6.0)*	
Physical activity	Less than recommended	1696 (40.7)	
	As recommended or more	2158 (51.7)	
	Missing	*318 (7.6)*	
BMI	Unhealthy BMI	1386 (33.2)	
	Healthy BMI	2641 (63.3)	
	Missing	*145 (3.5)*	
**Household characteristics**			
Type of household	One-person/parent household	353 (8.5)	123697 (15.3)
	Other	*3819 (91.5)*	684207 (84.7)
Marital status	Unmarried	2147 (51.5)	433383 (53.6)
	Married	2025 (48.5)	374521 (46.4)
Dissolution of marriage	Yes	58 (1.4)	14928 (1.8)
	No	4114 (98.6)	792976 (98.2)
Household size	≥6 persons	93 (2.2)	20412 (2.5)
	<6 persons	4079 (97.8)	757300 (93.7)

	Missing		*30192 (3.7)*
Youth support utilization	Yes	102 (2.4)	79473 (9.8)
	No	4070 (97.6)	728431 (90.2)
**Self-reported health**			
Perceived health status	Negative	*465 (11.1)*	
	Positive	3653 (87.6)	
	Missing	*54 (1.3)*	
Long-term illness	Yes	747 (17.9)	
	No	3362 (80.6)	
	Missing	*63 (1.5)*	
Restricted by health	Yes	724 (17.4)	
	No	3330 (79.8)	
	Missing	*118 (2.8)*	
**Healthcare expenditures and utilization**			
Overall healthcare expenditures	High	*824 (19.8)*	151443 (18.7)
	Low-average	3297 (79.0)	627116 (77.6)
	Missing	*51 (1.2)*	*29345 (3.6)*
GP expenditures	High	827 (19.8)	145166 (18.0)
	Low-average	3308 (79.3)	633247 (78.4)
	Missing	*37 (0.9)*	29491 (3.7)
Hospital expenditures	High	413 (9.9)	76523 (9.5)
	Low or none	3708 (88.9)	731381 (90.5)
	Missing	*51 (1.2)*	*0 (0.0)*
Medication use	High	428 (10.3)	92295 (11.4)
	Low or none	*3744 (89.7)*	715609 (88.6)
Addiction related care utilization	Yes	23 (0.6)	4466 (0.6)
	No	4149 (99.4)	803438 (99.4)
**Psychosocial characteristics**			
Mental healthcare utilization	Yes	228 (5.5)	50630 (6.3)
	No	3907 (93.6)	739093 (91.5)
	Missing	*37 (0.9)*	*18181 (2.3)*
Risk of depression or anxiety disorders	Moderate – high risk	1716 (41.1)	
	No or low risk	2256 (54.1)	

	Missing	*200 (4.8)*	
Loneliness	Feeling lonely	*1100 (26.4)*	
	Not feeling lonely	2719 (65.2)	
	Missing	*353 (8.5)*	
Feelings of control over life	Low	144 (3.5)	
	Moderate	2741 (65.7)	
	High	1006 (24.1)	
	Missing	*281 (6.7)*	
Mild intellectual disability	Yes	13 (0.3)	7187 (0.9)
	No	4159 (99.7)	800717 (99.1)
**Life-events**			
Crime suspect	Yes	*95 (2.3)*	35393 (4.4)
	No	4077 (97.7)	772511 (95.6)
Crime victim	Yes	874 (20.9)	171564 (21.2)
	No	3298 (79.1)	636340 (78.8)
Having been detained[a]	Yes	not shown	2592 (0.3)
	No	not shown	805312 (99.7)
History of frequent moving[b]	Yes	53 (1.3)	14081 (1.7)
	No	4119 (98.7)	793823 (98.3)
Loss of a family member	Yes	147 (3.5)	32839 (4.1)
	No	*4025 (96.5)*	775065 (95.9)
**Living conditions**			
Home ownership	Rented	990 (23.7)	264519 (32.7)
	Owner occupied	3099 (74.3)	531526 (65.8)
	Missing	*83 (2.0)*	*11859 (1.5)*
Motorized vehicle ownership	No	494 (11.8)	162579 (20.1)
	Yes	3678 (88.2)	645325 (79.9)
Proximity to General Practitioners' (GP) office	> 3 km	265 (6.4)	62040 (7.7)
	< 3 km	3847 (92.2)	740876 (91.7)
	Missing	*60 (1.4)*	*4988 (0.6)*
Liveability neighbourhood	Low-mediocre	273 (6.5)	87013 (10.8)
	High	3695 (88.6)	714817 (88.5)

	Missing	*204 (4.9)*	*6074 (0.8)*

a: Following guidelines of Statistics Netherlands, the data of some variables were rounded (parity) or not shown (having been detained) to prevent the disclosure of information about individuals.
b: Erratum: in the original paper of Molenaar et al. (12), there were inaccuracies in the reported percentages of the variable 'history of frequent moving', which consequently deviate from the values presented here.
Missing data are shown in italic.

Objective 1

Table B.2. Hyperparameters and thresholds used for RF, XGBoost and Lasso regression

	**Hyperparameters and thresholds**			
	threshold (average optimal)	lambda	tree depth	number of trees
Random Forest	0.37 (0.01)	NA	default	default
XGBoost	0.36 (0.04)	NA	2	51
Lasso regression	0.26 (0.04)	0.01 (0.003)	NA	NA

Results based on analyses among study population of 4172 women

Table B.3. Results of the RF and sensitivity analyses for the six separate folds

		**Metrics**				
		**AUC**	**F1-measure**	**precision**	**recall/ sensitivity**	**specificity**
**Random Forest**	**Average**	0.98 (0.00)	0.70 (0.03)	0.74 (0.06)	0.66 (0.04)	0.98 (0.00)
	Fold 1	0.98	0.68	0.69	0.67	0.98
	Fold 2	0.98	0.68	0.68	0.69	0.98
	Fold 3	0.98	0.70	0.74	0.67	0.98
	Fold 4	0.98	0.75	0.83	0.68	0.99
	Fold 5	0.98	0.72	0.74	0.69	0.98
	Fold 6	0.97	0.66	0.75	0.59	0.99
**XGBoost**	Mean	0.98 (0.00)	0.68 (0.04)	0.70 (0.02)	0.67 (0.08)	0.98 (0.00)
	Fold 1	0.98	0.68	0.68	0.69	0.98
	Fold 2	0.98	0.68	0.68	0.69	0.98
	Fold 3	0.98	0.61	0.70	0.55	0.98
	Fold 4	0.98	0.75	0.72	0.77	0.98
	Fold 5	0.98	0.66	0.74	0.60	0.99
	Fold 6	0.97	0.70	0.71	0.70	0.98
**Lasso regression**	Mean	0.98 (0.01)	0.68 (0.04)	0.67 (0.07)	0.70 (0.07)	0.98 (0.01)
	Fold 1	0.97	0.64	0.54	0.78	0.96
	Fold 2	0.98	0.71	0.65	0.77	0.98
	Fold 3	0.97	0.65	0.71	0.60	0.98
	Fold 4	0.98	0.74	0.74	0.73	0.98
	Fold 5	0.98	0.66	0.68	0.64	0.98
	Fold 6	0.97	0.69	0.70	0.67	0.98

Results based on analyses among study population of 4172 women

Table B.4. Average proportion of multidimensional vulnerability in the Netherlands over the years 2017 – 2021 for the three regression models

		2017	2018	2019	2020	2021
**RF**	average proportion	0.081	0.079	0.079	0.077	0.072
	SD	8.21E-05	2.65E-05	6.7E-05	5.93E-05	0.000105
**XGBoost**	average proportion	0.090	0.088	0.088	0.085	0.080
	SD	0.000105	0.000151	9.21E-05	7.95E-05	5.55E-05
**Lasso regression**	average proportion	0.100	0.098	0.098	0.097	0.091
	SD	0.000116	8.69E-05	7.05E-05	5.66E-05	9.52E-05

Table B.5. Complete cases

		2017	2018	2019	2020	2021
**RF**	proportion	0.086	0.084	0.083	0.080	0.075
**XGBoost**	proportion	0.095	0.092	0.091	0.087	0.081
**Lasso regression**	proportion	0.106	0.103	0.102	0.099	0.093

Results based on analyses among all unique pregnancies from 2017 – 2021 with complete data on all variables

Objective 2

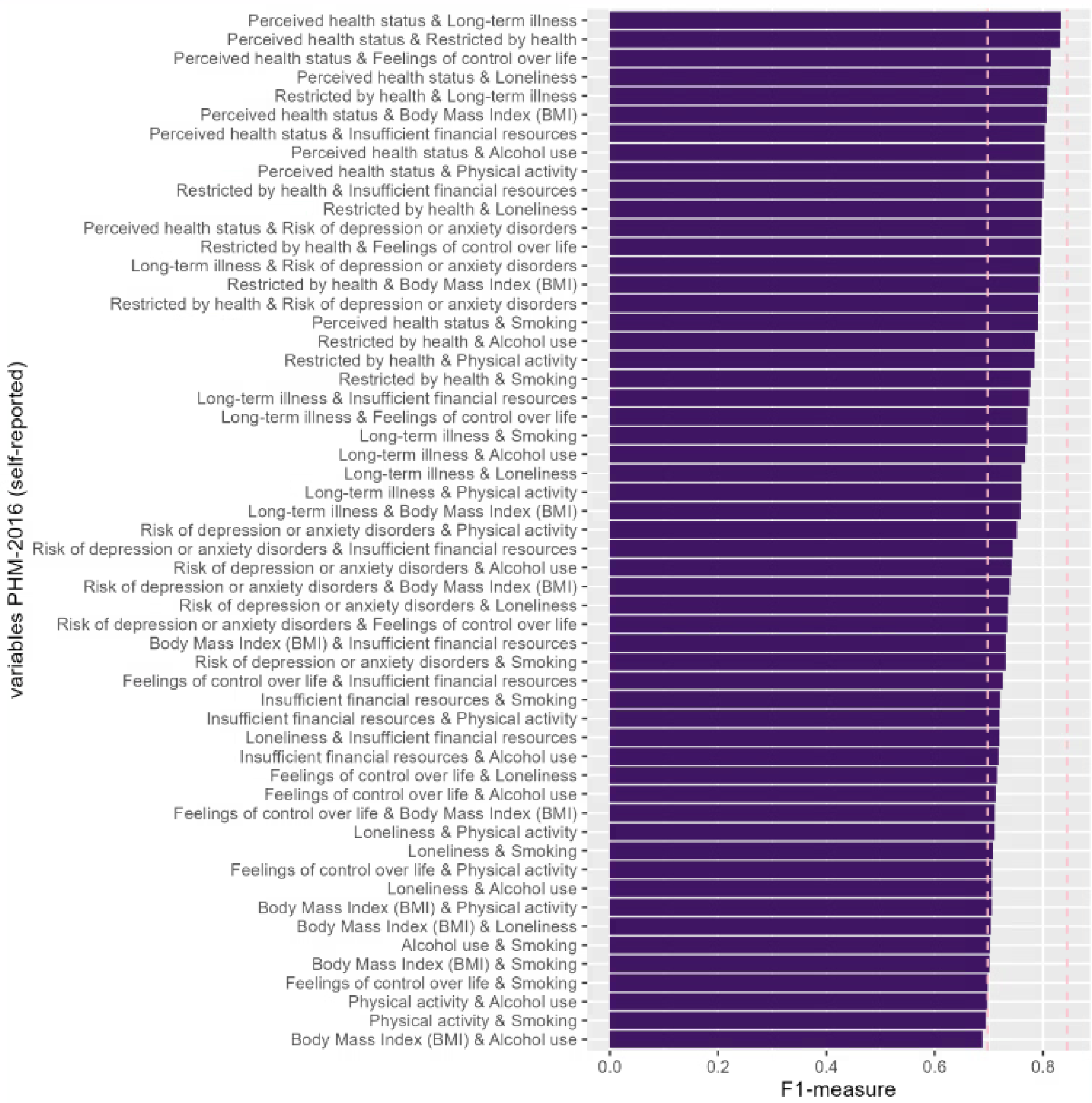

Figure B.1. Two variables on self-reported health, wellbeing and lifestyle added to the RF-models with solely routinely collected data.
Results based on analyses among study population of 4172 women.

Objective 3

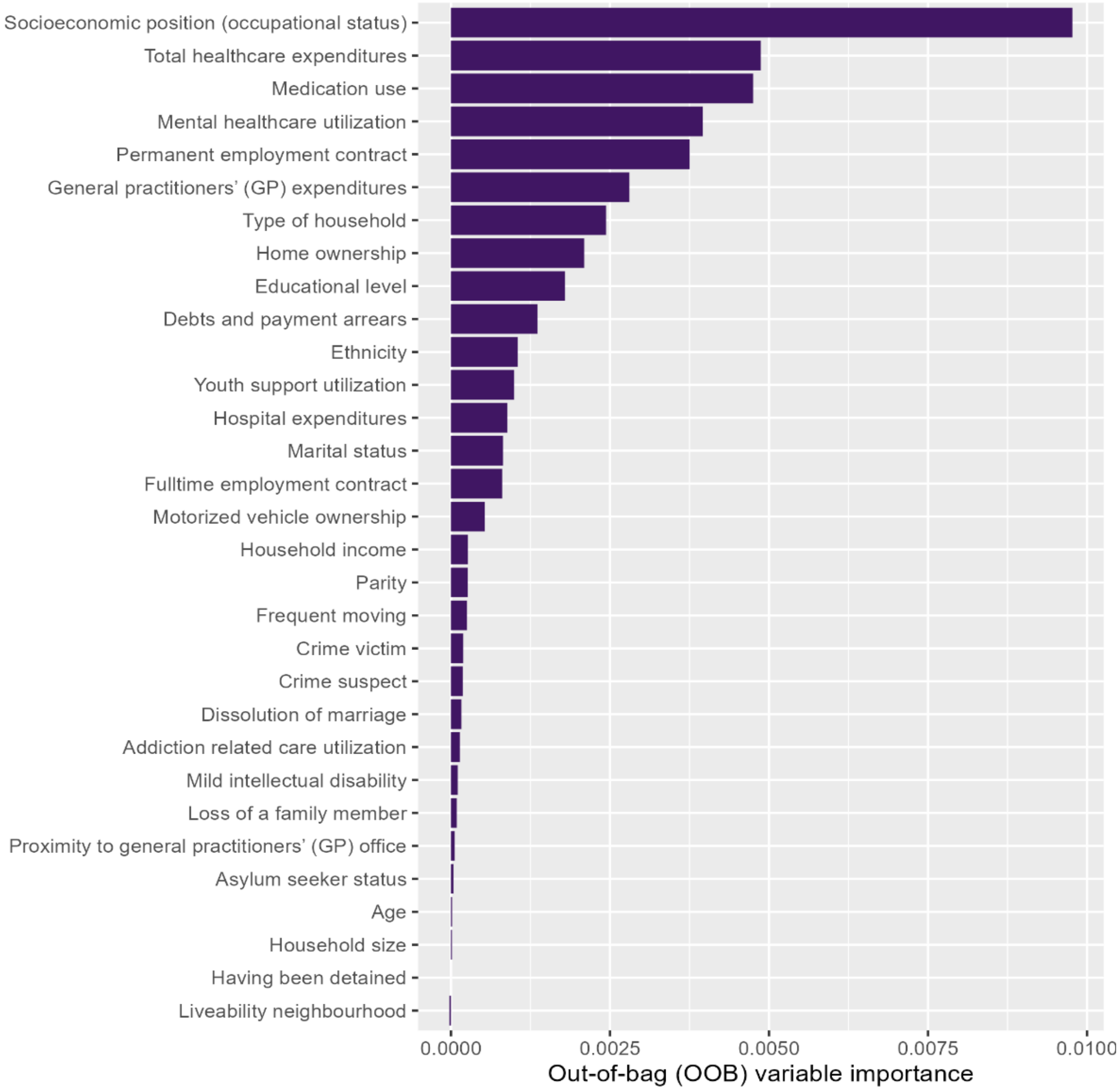

Figure B.2. Variable importance ranking of the RF-model for 'multidimensional vulnerability', using 31 variables (solely routinely collected data).

Results based on analyses among study population of 4172 women.

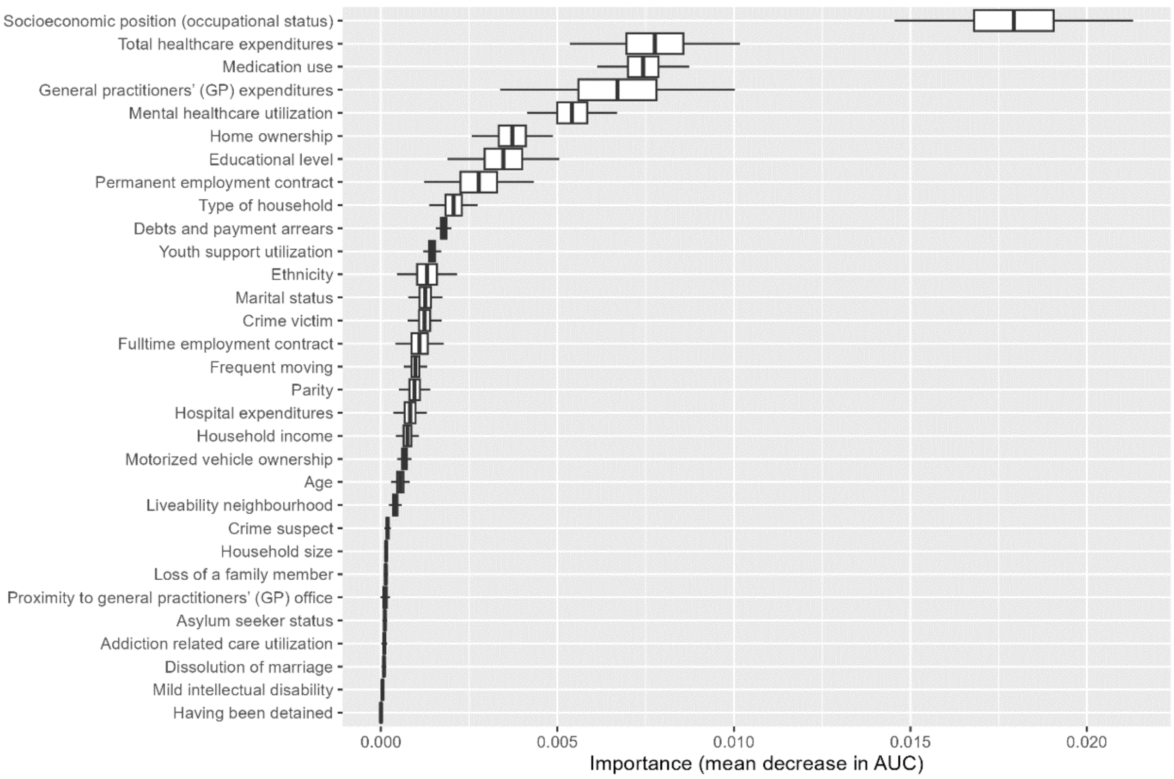

Figure B.3. Permutation importance ranking of the RF-model for 'multidimensional vulnerability', using 31 variables (solely routinely collected data)
Results based on analyses among study population of 4172 women

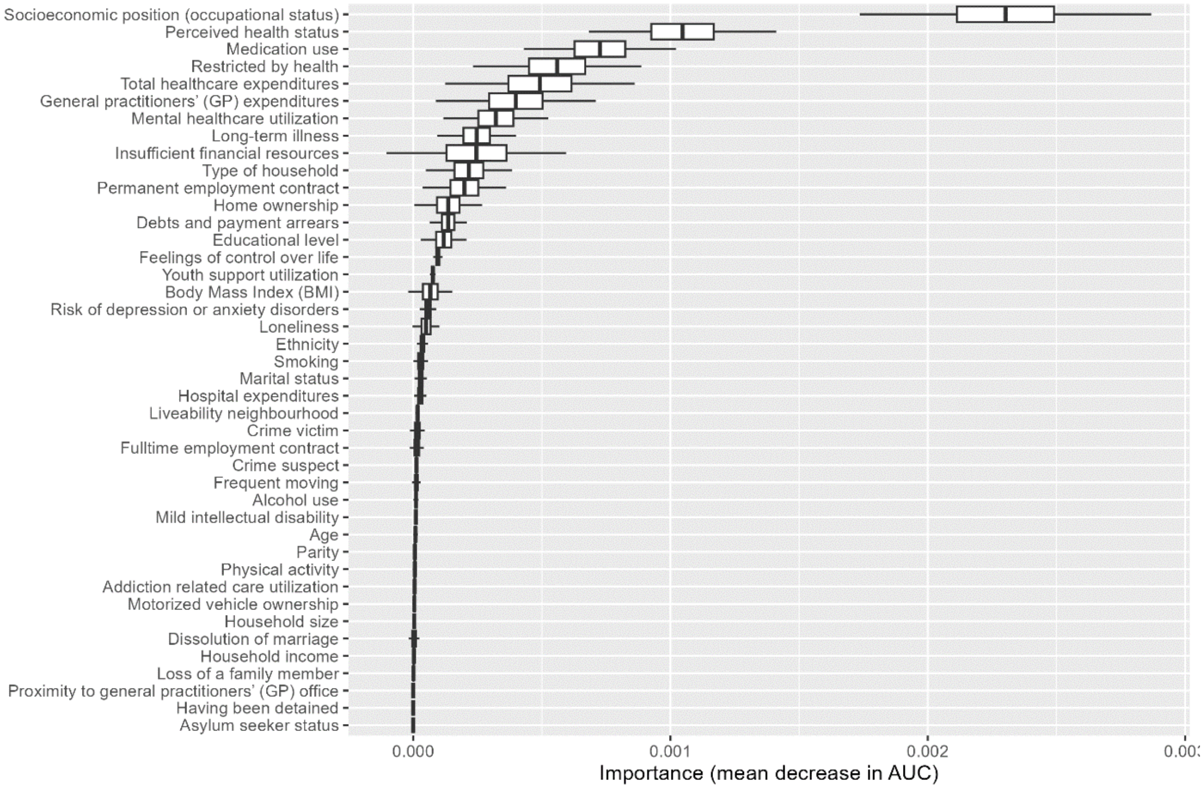

Figure B.4. Permutation importance ranking of the RF-model for 'multidimensional vulnerability', using the set of 42 variables (both routinely collected data and self-reported data)
Results based on analyses among study population of 4172 women

References

1. Chen T, Guestrin C, editors. Xgboost: A scalable tree boosting system. Proceedings of the 22nd acm sigkdd international conference on knowledge discovery and data mining; 2016.
2. Friedman J, Hastie T, Tibshirani R. Regularization paths for generalized linear models via coordinate descent. Journal of statistical software. 2010;33(1):1.
3. Wright MN, Wager S, Probst P. Package 'ranger' 2023 [Available from: https://mirror.las.iastate.edu/CRAN/web/packages/ranger/ranger.pdf.
4. Friedman J, Hastie T, Tibshirani R, Narasimhan B, Tay K, Simon N, Qian J. Package 'glmnet'. 2023.
5. Chen T, He T, Benesty M, Khotilovich V. Package 'xgboost' 2023 [Available from: https://cran.utstat.utoronto.ca/web/packages/xgboost/xgboost.pdf.
6. Breiman L. Random forests. Machine learning. 2001;45:5-32.
7. Janitza S, Celik E, Boulesteix A-L. A computationally fast variable importance test for random forests for high-dimensional data. Advances in Data Analysis and Classification. 2018;12:885-915.
8. Molnar C. Interpretable machine learning: Lulu. com; 2020.
9. Fisher A, Rudin C, Dominici F. All Models are Wrong, but Many are Useful: Learning a Variable's Importance by Studying an Entire Class of Prediction Models Simultaneously. J Mach Learn Res. 2019;20(177):1-81.
10. Friedman JH. Greedy function approximation: a gradient boosting machine. Annals of statistics. 2001:1189-232.
